# Prospects of *In vivo* Incorporation of Non-canonical Amino Acids for the Chemical Diversification of Antimicrobial Peptides

**DOI:** 10.3389/fmicb.2017.00124

**Published:** 2017-02-02

**Authors:** Tobias Baumann, Jessica H. Nickling, Maike Bartholomae, Andrius Buivydas, Oscar P. Kuipers, Nediljko Budisa

**Affiliations:** ^1^Biocatalysis Group, Department of Chemistry, Technische Universität Berlin (Berlin Institute of Technology)Berlin, Germany; ^2^Molecular Genetics Group, Department of Molecular Genetics, Groningen Biomolecular Sciences and Biotechnology Institute, Rijksuniversiteit Groningen (University of Groningen)Groningen, Netherlands

**Keywords:** antibacterial peptides, lantibiotics, non-canonical amino acids, orthogonal translation, aminoacyl-tRNA-synthetases, non-natural peptide variants, ribosomally synthesized and post-translationally modified peptides, nisin

## Abstract

The incorporation of non-canonical amino acids (ncAA) is an elegant way for the chemical diversification of recombinantly produced antimicrobial peptides (AMPs). Residue- and site-specific installation methods in several bacterial production hosts hold great promise for the generation of new-to-nature AMPs, and can contribute to tackle the ongoing emergence of antibiotic resistance in pathogens. Especially from a pharmacological point of view, desirable improvements span pH and protease resistance, solubility, oral availability and circulation half-life. Although the primary focus of this report is on ribosomally synthesized and post-translationally modified peptides (RiPPs), we have included selected cases of peptides produced by solid phase peptide synthesis to comparatively show the potential and impact of ncAA introduction. Generally speaking, the introduction of ncAAs in recombinant AMPs delivers novel levels of chemical diversification. Cotranslationally incorporated, they can take part in AMP biogenesis either through direction interaction with elements of the post-translational modification (PTM) machinery or as untargeted sites with unique physicochemical properties and chemical handles for further modification. Together with genetic libraries, genome mining and processing by PTM machineries, ncAAs present not a mere addition to this process, but a highly diverse pool of building blocks to significantly broaden the chemical space of this valuable class of molecules. This perspective summarizes new developments of ncAA containing peptides. Challenges to be resolved in order to reach large-scale pharmaceutical production of these promising compounds and prospects for future developments are discussed.

## Introduction

Constant isolation of new multidrug-resistant microbes affords a parallel development of new antimicrobial compounds for the treatment of infections. Today, important target species are MRSA, vancomycin-resistant enterococci (VRE), *Klebsiella pneumonia, Acinetobacter baumannii* and members of the genus *Pseudomonas* and *Salmonella*. Especially due to the different molecular architecture and mode of action, AMPs bear a great potential to tackle this global threat to public health with new compound scaffolds (Ferri et al., 2015). Development of novel antimicrobials employing modularization and alteration of genetic components (leader peptide, core and PTM genes) as well as genome mining have been reviewed recently (Montalbán-López et al., 2016). Besides PTM, a further level of combinatory options to diversify these peptides beyond the set of 20 canonical amino acids (cAAs) comes from the incorporation of ncAAs. Their potential for (re)shaping the physicochemical properties of AMPs is evident from polyketide and non-ribosomally synthesized peptide products, an important and large pool of ncAA-rich antimicrobial compounds (Walsh et al., 2013). Produced by all kingdoms of life and also part of the innate immune system of higher organisms, AMPs with antibacterial, anticancer and antiviral activities were discovered (Ageitos et al., 2016). With more than 3000 AMPs reported so far, we will focus on the potential and recent reports on ncAA-modified AMPs. For earlier studies (until 2013), readers are referred to Budisa (2013). Beyond the scope of this work, detailed information from more general as well as biomedical perspective including market potential, mode of action and production methods can be found in recent reviews (da Costa et al., 2015; Ageitos et al., 2016).

## Ribosomally Synthesized and Post-Translationally Modified Peptides

Antimicrobial peptides are mostly small cationic peptides comprised of 7–100 amino acids capable to interact with negatively charged microbial membranes (Ageitos et al., 2016). One special subgroup are RiPPs, which are genetically encoded and naturally produced by fungi and bacteria.

**FIGURE 1:**
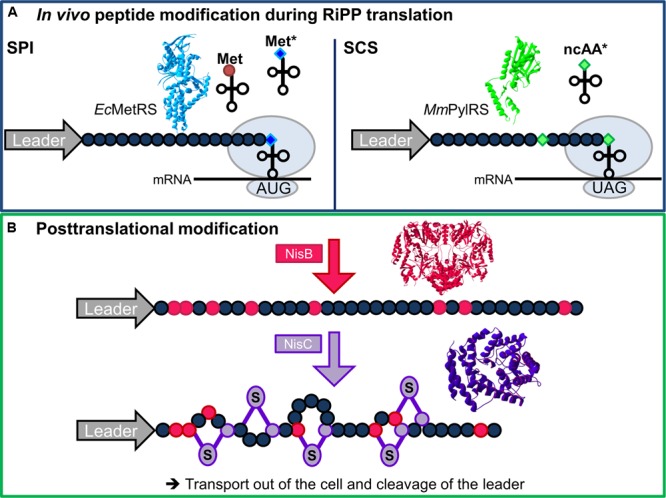
**Chemical diversification of AMPs by ncAAs. (A)** *In vivo* prepeptide modification by ncAAs during RiPP translation (core residues in dark blue circles). Incorporation of ncAAs (^∗^) can be achieved by two methods: Selective pressure incorporation (SPI, left) allows installation of isostructural variants of canonical amino acids, here as an example Met variants (blue diamonds) charged onto tRNA^Met^ by the endogenous *E. coli* methionyl-tRNA synthetase (MetRS, light blue, PDB ID **1PG2**; Crepin et al., 2003). The second method, stop codon suppression (SCS, right), requires co-expression of an orthogonal pair. A suppressor tRNA (here recognizing the amber stop codon UAG) is charged with the target ncAA (green diamonds) by its corresponding aminoacyl-tRNA synthetase (e.g., PylRS from *Methanosarcina mazeii*, depicted in green, PDB ID **2Q7H**; Kavran et al., 2007). **(B)** Posttranslational AMP modifications with the model lantibiotic nisin as chosen example. First, dehydration of certain prepeptide serine and threonine residues catalyzed by the dehydratase NisB (both in magenta, PDB ID **4WD9**; Ortega et al., 2015) yields dehydroalanines and dehydrobutyrines, respectively. Subsequent cyclization with Cys residues by the cyclase NisC (both purple, PDB ID **2G0D**; Li et al., 2006) affords the characteristic (methyl-)lantionine rings. The depicted elements are not true to scale. 3D structures of proteins rendered with Swiss PDB viewer version 4.1.0.

As illustrated for nisin in **Figure 1B**, RiPPs are initially produced as linear precursors composed of a leader and a core peptide region. Next, the inactive core undergoes vast chemical changes via PTM, e.g., dehydration, crosslinking, lanthionine formation and N-to-C cyclization. The N-terminal leader peptide serves three functions: directing the prepeptide to the modification enzymes, keeping the peptide inactive to protect the producer and steering secretion of the modified precursor peptide. Ultimately, a downstream protease cleaves the leader from the core, releasing the mature and subsequently active peptide, as described for the paradigm lantibiotic nisin. Via three principal mechanisms (da Costa et al., 2015), many RiPPs exhibit significant inhibitory activity against Gram-positive bacteria, e.g., *Streptococcus, Staphylococcus*, and *Bacillus* (Arnison et al., 2013).

## Natural PTMs to Diversify Physicochemical Properties of Peptides

Post-translational modification enzymes are valuable tools to modify and increase the diversity of existing peptides. Nisin, naturally produced by *Lactococcus lactis*, is the first described lantibiotic meanwhile used over 50 years in the food industry as a natural biopreservative without occurrence of bacterial resistance (Lubelski et al., 2008). Lantibiotics are characterized by the presence of (2*S*, 6*R*)-lanthionine or (2*S*, 3*S*, 6*R*)-3-methyllanthionine (Jung, 1991). These thioethers are post-translationally formed by dehydration of serine and threonine residues subsequently cross-linked via enzyme-catalyzed Michael addition of cysteine sulfhydryl groups. These intramolecular polycyclic configurations provide structural stability and resistance to protease degradation over linear peptide compounds (Rink et al., 2010). Besides lanthionine rings, other PTMs were discovered: e.g., formation of lasso peptides (Hegemann et al., 2015), glycocins (Norris and Patchett, 2016), linaridins (Rateb et al., 2015) or cyclic peptides such as the only recently described dikaritins (Ding et al., 2016). The high complexity of the molecules results in a very challenging chemical synthesis in large-scale production; e.g., total synthesis of nisin was achieved (Fukase et al., 1988), but with a crude yield of 0.003% before HPLC purification (Ongey and Neubauer, 2016). Total synthesis of lactocin *S* includes 71 reaction steps with a final yield of 10% (Ross et al., 2010). Biological production offers a feasible alternative because of high product concentrations, generation of the correct stereochemistry and less downstream processing steps. Substrate promiscuity of PTM enzymes (Oman and van der Donk, 2010) allows semisynthesis and combining them with hybrid leaders, enabling different modifications at the same core peptide (Montalbán-López et al., 2016). Consequently, search engines and databases for antimicrobials such as BAGEL3 (van Heel et al., 2013) or antiSMASH (Weber et al., 2015) are helpful tools for mining and designing new antibiotics (van Heel et al., 2016).

## Ribosomal Incorporation of ncAAs in RiPPs and Proteins

For recombinant peptide and protein production, two main methods enable the ribosomal incorporation of ncAAs (cf. **Figure 1A**): the SPI method and SCS.

The first methodology covers the residue-specific incorporation of ncAAs. Exploiting the substrate promiscuity of endogenous aaRSs and tolerance of the translation apparatus, many isostructural analogs can be installed in peptides and proteins. Utilizing auxotrophic host strains, high levels of exchange are commonly achieved. After depletion of the corresponding cAA, the ncAA is added and target gene expression is induced. Inevitably, residue-specific replacement leads to incorporation at all codons of the exchanged cAA. Consequently, all sites in the target gene and moreover in the host cell proteome are subjected to replacement. Site-directed mutagenesis allows removal of unwanted sites within the target, provided that replacements do not perturb structure and function. Regarding the proteome, despite quick stalling of cell division, significant amounts of modified target peptide or protein can frequently be produced (Budisa et al., 1995).

Pioneered by Schultz and coworkers, stop or quadruplet codon suppression constitutes the second option for ncAA incorporation (Wang et al., 2001; Anderson et al., 2004). o-pairs of a tRNA and a matching aaRS enable the site-specific installation of ncAAs. Via cycles of positive and negative screening/selection, ncAA-specific aaRS variants of *Methanocaldococcus jannaschii* TyrRS and *Methanosarcina barkeri*/*Methanosarcina mazei* PylRS can be isolated from gene libraries focusing on the active site architecture. Ideally, both components are fully orthogonal, i.e., not cross-reacting with host cell cAAs, tRNAs and aaRSs. Most commonly, the amber stop codon is employed. For its least frequently used stop codon, *Escherichia coli* tolerates the suppression by tRNAs aminoacylated with a large variety of ncAAs (Dumas et al., 2015).

Although *E. coli* presents the most commonly used host for ncAA incorporation, both methodologies have been employed in various hosts. Gram-positive bacterial species such as naturally poly-auxotrophic *Lactococcus lactis* strains are amenable for the force-feeding SPI approach (Chopin, 1993; Zhou et al., 2016). Two prominent examples of more complex organisms with proteome-wide ncAA labeling are the silkworm *Bombyx mori* (Teramoto and Kojima, 2015) and mice (Calve et al., 2016). Sophisticated *in vitro* translation systems have also been developed, allowing residue-specific (Worst et al., 2016) and site-specific (Chemla et al., 2015) ncAA incorporation - recently reviewed including display technologies for diversified natural products (Maini et al., 2016).

As introduced above, SPPS enables ncAA incorporation for which a variety of Fmoc-/Boc-protected ncAAs is commercially available.

## Potential of ncAAs in Antimicrobial Peptides

While proteins and especially the active sites of biocatalysts can be significantly reshaped using the set of 20 cAAs for first- and higher-shell mutations, the 3D-structure of peptides is more directly defined by the combination of primary structure and PTMs. With more than 150 ncAAs incorporated to date (Dumas et al., 2015), genetic code expansion introduces a drastically broadened set of chemistries into ribosomally produced peptides and proteins, e.g., by introducing atoms and functional groups rarely or never found in nature such as fluorine or organic azides. Photocaged residues allow spatiotemporal control over sidechain properties, which can serve as a prodrug activation mechanism. Installing chemical handles enables attachment of coupling partners by various reactions, where chemical or photoactivation can serve to install fluorescent dyes, glycans, PEGs, lipids or even other peptides and proteins (McKay and Finn, 2014). Consequently, ncAA incorporation offers unique physicochemical features over conventional peptide mutagenesis.

Using bacteria such as *E. coli* and *L. lactis* for recombinant production of AMPs confers several benefits. With well-established, efficient methods of genetic engineering, gene libraries of 10^5^–10^8^ variants can be created, offering multiple ways to alter precursor peptide and PTM machinery genes. Inducible/constitutive promoters, RBS libraries and high-/low-copy plasmid backbones offer combinatorial ways to control gene expression. The genetic diversity of such libraries can be sampled with good throughput for antimicrobial activity using indicator strain assays. Cheap media, high cell division rates and scalable production from microtiter plates to shake flasks and HCDC fermentation allow quick generation of peptide-producing biomass. Repeatedly, recombinant production could outperform the natural host (Ongey and Neubauer, 2016).

In contrast to SPPS, biosynthetic production of peptides and ncAA incorporation by SPI/SCS commonly work stereospecific, sparing the costly separation of racemic mixtures (Liu et al., 2011). Analogous to chemical strategies (Escano and Smith, 2015), changing the size and/or chemical nature of lantibiotic rings could be attempted via ncAAs.

## Antimicrobial Peptides Equipped With ncAas

Therapeutic use of ncAAs is an impressively broad field, comprising compounds of single amino acids to complex ncAA-modified protein structures. A comprehensive overview was recently published (Blaskovich, 2016). In this section, we will focus on ncAA-modified AMPs, their production and activities.

Since certain *Listeria* or *Brucella* species survive inside macrophages, they represent a special challenge for the development of antimicrobials. Proline-rich antibacterial peptides designed from PR-36 and bactenicin were equipped with ncAAs and fluorescein as tracking label. Depending on ncAA type and content, synthesized dual-action AMPs showed improved macrophage cell penetration and broad-spectrum activity against *Listeria, Brucella*, MRSA, *B. anthracis* and *Salmonella typhimurium*. Moreover, proteolytic resistance against trypsin was improved (Kuriakose et al., 2013). Later, tripeptides composed of histidine; arginine and lysine were modified with bulkier histidine analogs. From a panel of synthesized peptides, antifungal activities were obtained with no or acceptable cytotoxicity in cell culture assays (Mittal et al., 2016).

Protecting the expression host via a fusion protein, tritrpticin containing tryptophan analogs was produced in *E. coli* when the endogenous cAA synthesis was chemically inhibited. Antimicrobial activity and membrane permeabilization were retained after efficient (≥87.5%) fluorination of the three sites, which also enabled ^19^F NMR spectroscopy (Arias et al., 2016). Incorporation of tetra-substituted α-amino acids such as 1-aminocyclohexane carboxylic acid and 1-aminocyclopentane carboxylic acid provided peptides with activities against *Clostridium difficile* and *S. aureus* as well as Gram-negative species including *Klebsiella pneumonia, Salmonella enterica*, and *Acinetobacter baumannii* (Hicks, 2016). Additionally, some showed potent activity (IC_50_ < 10 μM) against cancer cell lines.

By a machine-based learning approach, eight AMPs containing ncAAs such as ornithine, norleucine, and homoarginine were obtained that inhibited *S. aureus* and *P. aeruginosa* (Wang et al., 2016). Mimicking a microbial membrane, MD simulations modeled the lipid bilayer interaction of the most potent peptide to shed light on the mode of action.

First studies illustrate the potential of RiPPs equipped with ncAAs by the two approaches described above. Both SPI and SCS were used to equip the lasso peptide capistruin with a total of seven ncAAs. With *N*_𝜀_-alloc-L-lysine installed via SCS, metathesis was conducted to covalently attach molecules using a ruthenium-based catalyst *in vitro* (Al Toma et al., 2015). Lasso peptide microcin J25 was successfully modified via PylRS-based SCS (Piscotta et al., 2015). Four *meta*-substituted phenylalanine derivatives were installed at four positions tested. Yields obtained for the 16 AMP variants depended on position and ncAA, and antimicrobial activity against *Salmonella newport* was retained.

Exploiting the substrate promiscuity of *L. lactis* TrpRS for SPI, tryptophan analogs with substitutions at position 5 were incorporated at four positions in nisin (Zhou et al., 2016). Likewise using the natural host instead of recombinant production, a thiocillin-producing *Bacillus cereus* strain was equipped with orthogonal translation (Luo et al., 2016). With tRNA and PylRS expression established, three ncAAs could be incorporated site-specifically. Fluorescent probe attachment by CuAAC and streptavidin-based capture of a biological target protein via photocrosslinking were achieved.

Besides influencing the microbial target spectrum and efficacy, ncAA modification also allows circumventing the necessity of a leader-cleaving protease. By incorporation of α-hydroxy acids into lacticin 481 and nukacin ISK-1 via a PylRS-based system, the leader was cleaved off by TFA and alkaline treatment (Bindman et al., 2015).

## Current Challenges in Recombinant Production of ncAA-Modified Peptides

Despite the promising features of ncAA-containing AMPs summarized above, some challenges remain to be overcome before this setup is ideal for large-scale synthesis of pharmaceuticals. For SPI, peptide precursor and PTM enzyme expression demands strict control over each part. One possibility is the utilization of two different inducible promoters (e.g., nisin- and Zn-inducible promoters in *L. lactis*) to uncouple modification enzyme expression which requires cAAs from RiPP synthesis which requires the ncAA (Zhou et al., 2016). Depending on the host, also the quality control during tRNA charging has to be considered. The phenylalanyl-tRNA synthetase of *Saccharomyces cerevisiae* discriminates badly between its natural substrate phenylalanine and four hydroxylated variants. However, after activation, these variants are not charged onto the tRNA, because the transfer is kinetically disfavored over the release from the active site (Moghal et al., 2016). This natural proofreading mechanism impedes unwanted synthesis of possible dysfunctional proteins. For SPI-based AMP production, it drastically reduces the yield, although ncAA activation kinetics are favorable.

For SCS, the genetic complexity of tRNA, aaRS, AMP precursor peptide and PTM enzyme expression demands well-balanced setups. Sophisticated combination of PylRS-based ochre (UAA) and *Mj*TyrRS-based amber (UAG) codon suppression enabled simultaneous incorporation of *𝜀*-tBoc-lysine and *p-*acetylphenylalanine (Chatterjee et al., 2013). For amber suppression in common *E. coli* production strains, the charged orthogonal tRNA competes with release factor 1 (RF-1), the endogenous protein facilitating translation. Furthermore, amber sites in the host genome lead to installation of the ncAA in various parts of the host proteome, which can limit cell growth and target production. For both *E. coli* K and B strains, genomically recoded organisms have been created by replacing all (Lajoie et al., 2013) or 95 (Mukai et al., 2015) genomic amber stop codons, respectively. Boosting amber suppression for poorly effective o-pairs and especially multi-site suppression, deletion of the otherwise essential RF-1 was achieved.

Isolated from genetic libraries for a defined ncAA, the aaRS substrate specificity commonly requires expression of a matching enzyme for each ncAA to be incorporated. Especially for sampling defined AMP positions with different ncAAs, polyspecific synthetases with high substrate promiscuity provide an interesting solution, with examples able to charge their tRNAs with up to 18 different ncAAs *in vivo* (Young et al., 2011; Guo et al., 2014).

Although proof-of-principle studies have shown that installation of multiple different ncAAs (e.g., combining SPI and SCS or amber stop with quadruplet codon suppression) can be achieved, this task remains challenging and optimizations are to be expected. General disadvantages of AMPs are limited stability at neutral or basic pH, limited oral availability, high susceptibility to renal clearance because of the high positive charge and also to proteolysis (Di, 2015; Escano and Smith, 2015). However, especially the latter problem can be faced with ncAA utilization. Incorporation of biphenylalanine and homoarginine into cationic tripeptides with reasonable activity against MRSA made them completely resistant against trypsin and increased stability up to 70-fold in stomach and 50-fold in liver in mouse whole organ extracts (Karstad et al., 2012).

One of the biggest disadvantages remain the costs for synthesizing RiPPs in reasonable amounts (Ongey and Neubauer, 2016). Still, the number of approved peptide drugs in recent years is rising and the need for new antimicrobials might also boost research funding (da Costa et al., 2015).

Last but not least, the combinatory options of 20 cAAs and more than 100 ncAAs affords elaborated design strategies for novel AMPs. Computer-based analysis and rational design are promising tools to create and improve ncAA-containing variants (He and He, 2016; Xiong et al., 2016). In combination with the 95 lanthipeptides described so far, sophisticated high-throughput screening methods are needed to reveal the best applications (Montalbán-López et al., 2012; Dischinger et al., 2014). For identified hits, thorough testing is needed, ideally including target strains of clinical relevance studied via standardized methods. Otherwise, results and efficacies remain difficult to compare and potent candidates may remain overlooked (Field et al., 2015; Ageitos et al., 2016).

## Perspective: Recombinant Production of ncAA-Modified Nisin Variants Via SPI

**FIGURE 2:**
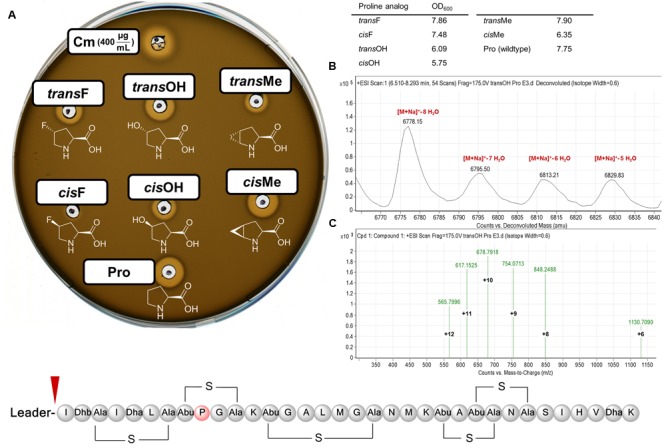
**Modification of nisin with ncAAs. (A)** Antimicrobial activity assay using novel nisin variants produced by recombinant expression and SPI using ncAA analogs of proline. *E. coli* expression samples (harvested cell densities tabulated as OD_600_) were normalized and tested for inhibition of the Gram-positive indicator strain *L. lactis* NZ9000 carrying plasmid pNG *nisPT* for cleavage of the AMP leader (Khusainov and Kuipers, 2013). Cm: 400 μg/mL chloramphenicol (antibacterial control); ncAAs used for SPI are abbreviated above and depicted below the corresponding wells: *cis*/*trans*-4-fluoroproline ((4*S*/*R*-F)Pro), *cis*/*trans*-4-hydroxyproline ((4*S*/*R*-OH)Pro), *cis*/*trans*-methanoproline, proline (wild-type control). See Supplementary Information for assay details. Nisin structure including (methyl)lanthionine rings and NisP cleavage site (red triangle) depicted at the bottom, highlighting position of proline 9 (red circle) targeted for modification by ncAAs. **(B)** MS deconvolution chromatogram for recombinant nisin containing *trans*-4-hydroxyproline. Calculated masses (Da): [M+Na]^+^ – 8 H_2_O = 6779.21, [M+Na]^+^ – 7 H_2_O = 6795.21, [M+Na]^+^ – 6 H_2_O = 6813.21, [M+Na]^+^ – 5 H_2_O = 6831.21 **(C)** Compound spectrum for charged species of [M+Na]^+^ – 8 H_2_O.

For the class I model lantibiotic nisin, we targeted the core peptide proline for replacement by ncAAs. Also found in nisin Z and Q, subtilin, ericin A and S, epidermin as well as gallidermin (Rink et al., 2005), the conserved residue is crucial for activity (Rink et al., 2007). Besides structure of the pre- and/or propeptide and ultimately antimicrobial activity, replacement by proline analogs should affect the rigidity (Kubyshkin et al., 2016) of ring B. Inspired by SPI using *L. lactis* (Zhou et al., 2016), recombinant nisin production was conducted and combined with SPI using a proline-auxotrophic *E. coli* strain. *L. lactis* expressing and secreting NisP, thus activating the nisin variants recombinantly produced by *E. coli*, was used for activity determination. Evidently, growth inhibition could be observed for most of the chosen proline analogs (**Figure 2**). Low ncAA toxicity is evident from the production strain reaching high culture densities. Without ncAA addition, *E. coli* cell densities stay low after induction and no activity can be observed (data not shown). With NisB and NisC activity evident from this assay, it should be noted that both enzymes carry 23 and 5 proline residues, respectively, which become likewise modified during SPI. For the two most active compounds, *trans*-4-hydroxyproline and *trans*-4-fluoroproline, peptides were affinity-purified. ESI-MS analysis confirmed ncAA installation. As before (Shi et al., 2011), multiple dehydration extents are observed. Consequently, our data show that recombinant production of ncAA-modified bioactive nisin is feasible.

Emphasizing the effects of prolines in AMPs, proline replacement of N20 in the hinge region of nisin improved antimicrobial activity against MRSA (Field et al., 2008).

## Outlook

Current literature shows that diversification of AMPs harbors great potential. As for conventional mutagenesis, structure-function studies with ncAAs reveal new-to-nature peptide products with novel properties and chemical functionalities.

Certainly, the complexity of recombinant AMP expression including a functional PTM machinery and SPI/SCS-based ncAA incorporation (cf. **Figure 1**) presents a challenging task for bioprocess and production strain engineering. With commonly high prices for chiral ncAAs, cost-efficiency can be improved by metabolic engineering of bacterial production strains to produce ncAAs from cheap precursors (Ma et al., 2014; Anderhuber et al., 2016). Optimizing o-pair efficiency and expression can also reduce the amounts of ncAA needed or improve production yields. Even for sophisticated SCS setups, efficiency improvements are to be expected in the near future (Zheng et al., 2016).

For residue-specific incorporation, production strains could be streamlined to ncAA incorporation as shown recently for an *E. coli* strain adapted to L-β-(thieno[3,2-*b*]pyrrolyl)alanine used to produce the correspondingly modified lantibiotic lichenicidin (Kuthning et al., 2016). As an alternative to recombinant production, orthogonal translation can be introduced into native producers, as shown for *B. cereus* (Luo et al., 2016). This way, AMP production could benefit from well-balanced expression and activity levels of precursor and PTM machinery genes.

Provided that activity can be transferred, combination of PTM enzymes from different AMPs offers additional diversity for the generation of novel AMPs as recently shown for D-alanine generation in dermorphin (Huo and van der Donk, 2016). In this direction, *in vitro* AMP production using ncAA- modified precursor peptides and purified PTM enzymes is feasible. Together with the rich lantibiotic diversity and their PTM genes in nature, ncAA incorporation provides an arsenal for AMP hypermodification.

## Author Contributions

TB, JN, and MB drafted the manuscript. TB and JN designed, performed and evaluated the experiments of the corresponding section of this Perspective. AB designed and constructed the expression plasmids for recombinant nisin production. AB, OK, and NB contributed to revise the manuscript to reach the final version. All authors read and approved the final manuscript.

## Conflict of Interest Statement

The authors declare thatthe research was conducted in the absence of any commercial or financial relationships that could be construed as a potential conflict of interest.

## Abbreviations

aaRS: aminoacyl-tRNA synthetase
AMP: antimicrobial peptide
cAA: canonical amino acid
MRSA: methicillin-resistant *Staphylococcus aureus*
ncAA: non-canonical amino acid
o-pair: orthogonal pair
PTM: post-translational modification
RiPPs: ribosomally synthesized and post-translationally modified peptides
SCS: stop codon suppression
SPI: selective pressure incorporation
SPPS: solid phase peptide synthesis
