# Supplementary material for: Prospects of In vivo Incorporation of Non-canonical Amino Acids for the Chemical Diversification of Antimicrobial Peptides
Source: Front Microbiol. 2017 Feb 2;8:124. doi: 10.3389/fmicb.2017.00124 (PMC5288337; doi:10.3389/fmicb.2017.00124)
Supplement: Supplementary file 1 [file Data_Sheet_1.DOCX]

Supplementary Material

Prospects of *in vivo* incorporation of noncanonical amino acids for the chemical diversification of antimicrobial peptides

Tobias Baumann^1^*, Jessica H. Nickling^1#^, Maike Bartholomae^2#^, Andrius Buivydas^2^, Oscar P. Kuipers^2^, Nediljko Budisa^1^*

*: Correspondence: [tobias.baumann@tu-berlin.de](mailto:tobias.baumann@tu-berlin.de), [nediljko.budisa@tu-berlin.de](mailto:nediljko.budisa@tu-berlin.de)

#: JHN and MB contributed equally to this work.

# Materials and methods

## Expression and purification of nisin variants containing proline analogs

The proline auxotrophic strain *E. coli* MG1655 Δ*proBA*::frt Δ*proC*::frt (DE3) was transformed with pET-3a *nisA* and pRSFDuet-1 *nisBC* plasmids. Cells were cultured overnight at 37 °C in LB medium containing ampicillin, kanamycin and 1 % (w/v) glycerol. 1 L of fresh medium was inoculated (1 % v/v) and incubated to a cell density of OD_600_ = 0.5. Subsequently, cells were washed and finally resuspended in 500 mL with new minimal medium (NMM, (Budisa et al., 1995)) lacking proline and containing antibiotics as well as glycerol (1 % v/v). After 1 h at 30 °C, the culture was divided into equal parts and adjusted to 1 mM IPTG and 1 mM proline analogs (*cis*/*trans*-4-fluoroproline ((4*S*/*R*-F)Pro), *cis*/*trans*-4-hydroxyproline ((4*S*/*R*-OH)Pro), *cis*/*trans*-4-methanoproline). Cells were grown overnight at 28 °C. Pelleted cells were resuspended in 4 M GdmCl, 25 mM Tris, 300 mM NaCl pH 7.5, sonicated and centrifuged at 15,000 g for 40 min at 4 °C. The supernatant containing nisin variants with N-terminally His-tagged leader was loaded onto a 1 ml Ni-NTA affinity column (HisTrap FF Crude, GE Healthcare). Washing and elution were performed with buffers containing 20 mM or 250 mM imidazole, respectively.

## LC-MS analysis

Peptide samples were analyzed via LC-ESI-TOF-MS using a QTOF 6530 instrument coupled to a 1260 HPLC system (Agilent). A 2.1x100 mm, 3 micron C5 column (Supelco analytical, Sigma-Aldrich) was run at a flow rate of 0.3 ml min^-1^ with a 20 min gradient from 5 % ACN 0.1% formic acid to 80% acetonitrile 0.1% formic acid in water. Spectra were analyzed using MassHunter Qualitative Analysis v. B.06.00 (Agilent) and maximum entropy deconvolution.

## Antimicrobial activity test

The indicator strain *L. lactis* NZ9000 carrying plasmid pNG *nisPT* (Khusainov and Kuipers, 2013) was cultured overnight at 30 °C in M17 broth with 1 % (w/v) glucose and 5 µg/ml chloramphenicol (Cm). Next, fresh medium was inoculated and incubated to a cell density of OD_600_ 0.4-0.6. The culture (2 % v/v) was added into molten GM17-agar containing Cm and poured into plates.

*E. coli* expression culture samples (1 mL) were centrifuged at 3000 g for 3 min. Pellets were resuspended in 500 µl sodium phosphate buffer (50 mM, pH 7.4) and sonicated on ice. Cellular debris was removed by centrifugation. Cell extract supernatants were diluted and normalized to an OD_600_ = 0.6 relative to the harvested cell density. 50 µl of each diluted sample were transferred into 7 mm holes of the indicator plate subsequently incubated overnight at 30 °C. Cm at 400 µg/mL served as antibacterial control compound.

# Supplementary data

## MS-data for recombinant nisin containing *trans*-4-fluoroproline


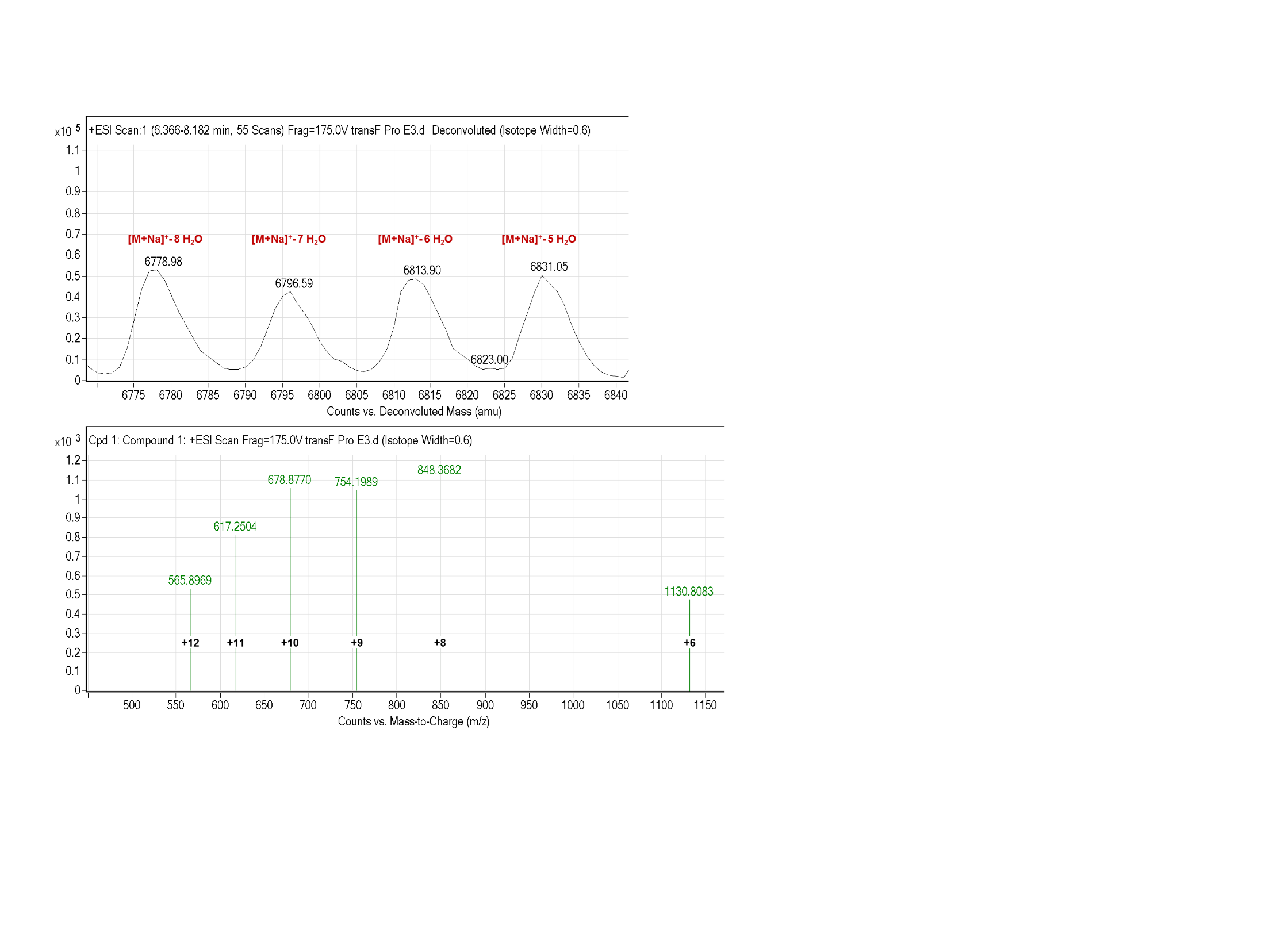


**Supplementary Figure 1.** **(A)** MS deconvolution chromatogram for recombinant nisin containing *trans*-4-fluoroproline. Calculated masses: [M+Na]^+^ - 8 H_2_O = 6779.21, [M+Na]^+^ - 7 H_2_O = 6797.21, [M+Na]^+^ - 6 H_2_O = 6815.21, [M+Na]^+^ - 5 H_2_O = 6833.21 **(B)** Compound spectrum for species [M+Na]^+^ - 8 H_2_O.
